# Loss of transforming growth factor-beta 2 leads to impairment of central synapse function

**DOI:** 10.1186/1749-8104-3-25

**Published:** 2008-10-14

**Authors:** Katharina Heupel, Vardanush Sargsyan, Jaap J Plomp, Michael Rickmann, Frédérique Varoqueaux, Weiqi Zhang, Kerstin Krieglstein

**Affiliations:** 1 Department of Neuroanatomy, University of Goettingen, Kreuzbergring 36, 37075 Goettingen, Germany; 2 Center for Molecular Physiology of the Brain (CMPB), University of Goettingen, Germany; 3 Department of Neurophysiology, University of Goettingen, Humboldtallee 23, 37073 Goettingen, Germany; 4 Departments of Neurology and Neurophysiology, Leiden University Medical Centre, 2300 RC Leiden, the Netherlands; 5 Max-Planck-Institute of Experimental Medicine, Hermann-Rein-Strasse 3, 37075 Goettingen, Germany; 6 Institute for Anatomy and Cell Biology, Department of Molecular Embryology, University of Freiburg, Albertstrasse 17, 79104 Freiburg, Germany

## Abstract

**Background:**

The formation of functional synapses is a crucial event in neuronal network formation, and with regard to regulation of breathing it is essential for life. Members of the transforming growth factor-beta (TGF-β) superfamily act as intercellular signaling molecules during synaptogenesis of the neuromuscular junction of *Drosophila *and are involved in synaptic function of sensory neurons of *Aplysia*.

**Results:**

Here we show that while TGF-β2 is not crucial for the morphology and function of the neuromuscular junction of the diaphragm muscle of mice, it is essential for proper synaptic function in the pre-Bötzinger complex, a central rhythm organizer located in the brainstem. Genetic deletion of TGF-β2 in mice strongly impaired both GABA/glycinergic and glutamatergic synaptic transmission in the pre-Bötzinger complex area, while numbers and morphology of central synapses of knock-out animals were indistinguishable from their wild-type littermates at embryonic day 18.5.

**Conclusion:**

The results demonstrate that TGF-β2 influences synaptic function, rather than synaptogenesis, specifically at central synapses. The functional alterations in the respiratory center of the brain are probably the underlying cause of the perinatal death of the TGF-β2 knock-out mice.

## Background

Proper synapse formation represents the basis for the regulation of vital functions and motor activity as well as for higher brain functions such as perception, learning, memory and cognition. Factors that influence the morphological development of synapses and that are capable of acutely modulating synaptic activity include neurotrophins such as nerve growth factor or the brain-derived neurotrophic factor [1]. Other extracellular signaling factors, such as Wnt-proteins and members of the transforming growth factor-beta (TGF-β) family of proteins, are regarded as target-derived signals in synaptogenesis of the invertebrate neuromuscular junction (NMJ) [2,3]. TGF-βs comprise a large superfamily of proteins with various functions in development and differentiation of the organism [4]. They signal through type I and II serine/threonine-kinase receptors (TβRI and TβRII) and the downstream signaling involves either smad-dependent or non-smad cascades, such as the ERK, JNK and p38 mitogen-activated protein kinase (MAPK) pathways [5]. The three isoforms of TGF-β proper, TGF-β1, TGF-β2 and TGF-β3, serve functions that range from the control of cell proliferation, cell adhesion, and extracellular matrix production, to differentiation, survival and death of cells [4]. Evidence that they are also involved in the development and function of synapses comes from studies in a number of organisms: in chick ciliary ganglionic neurons, target-derived TGF-β1 regulates the developmental expression and translocation of Ca^2+^-activated K^+ ^(K_Ca_) channels *in vitro *and *in vivo *[6,7]. TGF-β1 also has a prominent role in long-term synaptic facilitation in isolated *Aplysia *sensory ganglia [8]: within minutes, TGF-β1 stimulates MAPK-dependent phosphorylation of synapsin, which in turn modulates synapsin distribution, and results in a reduced magnitude of synaptic depression [9]. TGF-β2 modulates synaptic efficacy and plasticity in dissociated rat hippocampal neurons [10].

Mouse mutants with a deletion of a single TGF-β isoform have been generated and analyzed [11-13]. In the case of the TGF-β2 knock-out (KO) mice, the animals exhibit severe developmental deficits and die perinatally. Sanford and co-workers [12] suggest either pulmonal, cardiovascular or neuromuscular failure as the underlying defect. Despite the fact that the deletion of TGF-β2 results in a phenotype that is comparable with other mouse mutants in which defects in the generation of the NMJ have been identified [14-16], the neuromuscular system has not been investigated so far. At the mammalian NMJ, TGF-β2 takes up a special role among the three TGF-β ligands. Even though all three isoforms and their receptors are expressed by motoneurons, muscle and Schwann cells [17,18], TGF-β2 is the only isoform differentially regulated during muscle development and finally localized subsynaptically [19]. This situation resembles the localization pattern of another TGF-β-superfamily member, the bone morphogenetic protein (BMP)-7 homologue, glass-bottom-boat (gbb), and its type II receptor, wishful thinking (wit), in *Drosophila*. Interestingly, gbb [20,21], wit [22,23], the type I receptors, thickveins (tkv) and saxophone (sax) [24,25], and the smad-homologues, mothers against decapentaplegic (mad) and medea (med) [24,25], have convincingly been shown to regulate the development of the NMJ in fly larvae. The mutation of wit leads to defects in the ultra-structure of the NMJ, to a loss of the number of synaptic endplates and to a reduced frequency of spontaneous neurotransmitter release. Similar observations have been made in flies with mutations in gbb, tkv, sax, and in mad and med.

Together, increasing evidence suggests that TGF-β2 may be involved in synaptogenesis, modulation of synaptic transmission, synaptic plasticity and especially in shaping the mammalian NMJ. However, a systematic analysis addressing the role of TGF-β2 in synaptogenesis and neuronal network function in mammals *in vivo *is still lacking even though a putative role has been discussed in the literature [3,26]. Our work is the first example of TGF-β2 acting as an important regulator of central nervous system synaptic function *in vivo *in the pre-Bötzinger-complex (preBötC), which is part of the respiratory neuronal network in the brainstem. While the peripheral execution of breathing at diaphragm muscle NMJs is intact, TGF-β2 KO mice most likely die from respiratory failure due to impaired neurotransmission within the neuronal network of the preBötC.

## Results

### TGF-β2 KO mice do not display rhythmic respiratory activity

**Figure 1:**
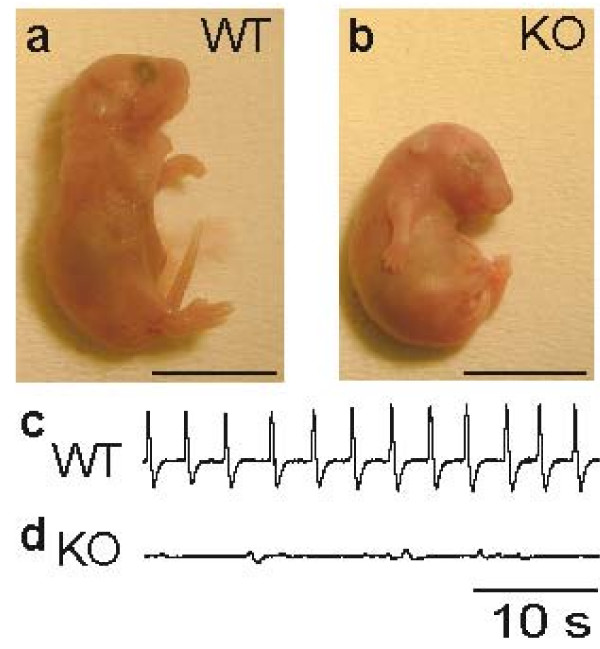
**Phenotype and breathing activity in wild-type (WT) littermates and transforming growth factor (TGF)-β2 knock-out (KO) mice at embryonic day 18.5.(a, b).**In comparison to WT embryos (a), TGF-β2 KO mice (b) display a normal gross anatomy, but a hunched posture and no voluntary movement.(c, d) Analysis of breathing activity showed a rhythmic pattern in WT embryos (c) but respiratory failure in KO mice (d). Scale bar, 1 cm (a, b).

At embryonic day (E) 18.5, TGF-β2 KO mice (Figure 1b) were smaller than wild-type (WT) littermates (Figure 1a) and displayed a hunched posture. Following cesarean section, TGF-β2 KO mice were first alive but neither showed voluntary movement nor any respiratory activity as recorded by whole-body plethysmography (Figure 1d). Within 30 minutes, TGF-β2 KO mice became cyanotic and died. In contrast, WT littermates did show rhythmic respiratory activity in the majority of animals that were examined (Figure 1c).

### NMJs of TGF-β2 KO mice develop normally and show successful synaptic transmission

The diaphragm is the peripheral executer of breathing activity and, therefore, its development and functionality have to be established prior to birth. Moreover, the NMJ is a well established model for studying synaptogenesis [27]. We thus studied the development and the function of the NMJs formed between the phrenic nerve and the diaphragm muscle in embryonic WT and KO mice at E18.5.

**Figure 2:**
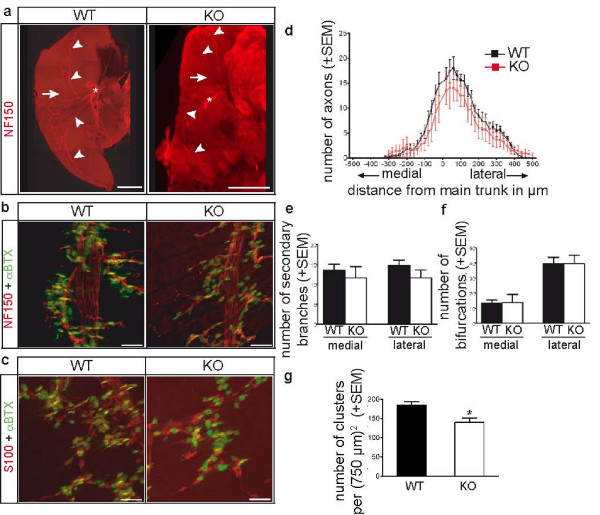
**Immunohistological analysis of the innervation of the diaphragm and of the morphology of neuromuscular junctions (NMJs).** (a) Innervation of the diaphragm by the phrenic nerve, labeled with anti-neurofilament (NF)150-antibody (red), in embryonic wild-type (WT) and knock-out (KO) mice. The phrenic nerve enters the diaphragm (asterisk) and splits up into three primary branches (arrowheads), from which secondary branches (arrow) branch off. Scale bar, 1 mm. (b) Acetylcholine receptors (AChRs), labeled with α-bungarotoxin (α-BTX; green), are clustered on the muscle surface and are innervated by secondary branches of the phrenic nerve in WT and KO diaphragms. Scale bar, 40 μm. (c) Schwann cells, labeled with anti-S100-antibody (red), accompany the phrenic nerve and can be found in close apposition to AChR clusters (green) in WT and KO animals. Scale bar, 40 μm. (d-f) Analysis of the branching pattern of the phrenic nerve in WT and KO diaphragms, which was quantified by determining the number of axon branches that crossed equidistant lines parallel to the main nerve trunk (d). The number of all secondary branches exiting the main nerve trunk on either side (e) and the overall number of bifurcations on either side of the main nerve trunk were also determined (f). Bars represent the mean ± standard error of the mean (SEM) of n = 5 WT or n = 4 KO animals. (g) The number of AChR clusters was determined in diaphragms of WT and KO mice. Bars represent the mean ± SEM of n = 6 WT and 6 KO animals. *P < 0.05.

To study whether the loss of TGF-β2 leads to changes in the morphology of the NMJ, we first analyzed the branching pattern of the phrenic nerve on the diaphragm to see whether its fasciculation or the branching on the muscle surface were altered. As shown in Figure 2a in the lower magnifications, the phrenic nerve split up into three primary branches in KO as in WT mice. At higher magnifications it can be seen that the fasciculation of the phrenic nerve in KO mice was also comparable to WT, that is, bundling into parallel fibers perpendicular to the length of the muscle fibers (Figure 2b). To compare the branching pattern of the motoneuron axons of WT and KO littermates, confocal images of anatomically comparable regions of the diaphragm were taken and analyzed as described by others [28]. We found no difference in the branching pattern (Figure 2d), the number of branches (medial WT: 14 ± 2, n = 5; medial KO: 12 ± 3, n = 4, not significant (NS), Student's *t*-test; lateral WT: 15 ± 1, n = 5; lateral KO: 12 ± 2, n = 4, NS, Student's *t*-test; Figure 2e) or the number of bifurcations (medial WT: 13 ± 2, n = 5; medial KO: 14 ± 5, n = 4, NS, Student's *t*-test; lateral WT: 40 ± 4, n = 5; lateral KO: 40 ± 6, n = 4, NS, Student's *t*-test; Figure 2f). In both WT and TGF-β2 KO mice, there were more branches and bifurcations on the lateral side of the main nerve trunk.

On the postsynaptic side, acetylcholine receptors (AChRs) aggregate in clusters at the sites of synaptic contact and the AChR clusters typically form a discrete endplate band in the center of the muscle fibers on each diaphragm side. In WT as in TGF-β2 KO diaphragms, AChR clusters formed (as revealed by α-bungarotoxin-labeling; Figure 2b,c) and were arranged in a central endplate band. We determined the width of the endplate band as described by others [29] and found that the average half-maximal width did not differ in WT and KO mice (WT: 132.7 ± 18.50 μm, n = 5; KO: 132.4 ± 18.22 μm, n = 5, NS, Student's *t*-test). The AChR-clusters were innervated by nerve processes to the same extent in TGF-β2 KO as in WT animals, which is also reflected in the findings that neither the branching pattern of the phrenic nerve nor the half-maximal width of the central endplate band were changed in TGF-β2 KO diaphragms. We also counted AChR clusters per visual field (750 × 750 μm^2^). This revealed a significant loss of about 25% of the clusters in TGF-β2 KO (WT: 185 ± 9, n = 6; KO: 140 ± 12, n = 6, *P *< 0.05; Figure 2g). Schwann cells accompany the phrenic nerve and terminal Schwann cells cap the NMJ. As shown in Figure 2c by immunohistochemical staining with S100 antibody, Schwann cells were present in TGF-β2 KO mice as they were in WT littermates. They were located in close relationship to the AChR clusters and accompanied the phrenic nerve. Taken together, apart from the loss of AChR clusters, the development of NMJs of TGF-β2 KO mice was similar to WT animals.

**Figure 3:**
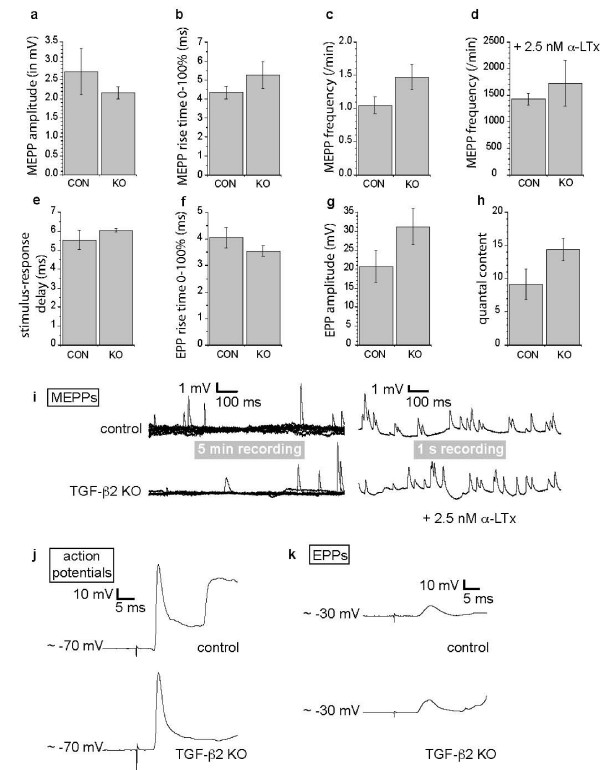
**Electrophysiological assessment shows normal synaptic transmission at transforming growth factor (TGF)-β2 knock-out (KO) neuromuscular junctions (NMJs). **(a-h) Bar graphs display the group mean values ± standard error of the mean (n = 3 and 5 embryos for the TGF-β2 KO and control (CON) groups, respectively; data from 3–20 NMJs sampled per muscle). (a) Amplitude, (b) rise-time and (c) frequency of miniature endplate potentials (MEPPs) are unchanged (*P *= 0.52, 0.23 and 0.11, respectively). (d) MEPP frequency evoked by 2.5 nM α-latrotoxin (α-LTx) is normal (*P *= 0.48). (e) Similar delay between nerve stimulation and start of the recorded postsynaptic response, either an action potential or an endplate potential (EPP; *P *= 0.49). (f) Rise time and (g) amplitude of EPPs are normal, as well as (h) the calculated quantal content, that is, the number of transmitter quanta released upon one nerve impulse. (i) Example traces of MEPP recordings in normal Ringer's medium (left panel, showing all MEPPs encountered during a 5 minute recording period) and in the presence of 2.5 nM α-LTx (right panel, 1 s recorded). (j) Examples of recorded muscle fiber action potentials following from a single nerve stimulation. The ensuing contraction of the impaled fiber (and neighboring fibers) leads to the contraction artifact visible in the recording trace just after the action potential. (k) At fibers that were allowed to depolarize to around -30 mV by waiting for some time, a single nerve stimulation evoked an EPP. Example traces of EPPs with similar characteristics recorded in TGF-β2 KO and control fibers. Contraction of neighboring fibers is visible as an artifact on the signal, starting just after the EPP.

In order to investigate whether neuromuscular synaptic dysfunction contributed to the observed lack of breathing, we studied synaptic transmission at NMJs of dissected diaphragm muscles of three TGF-β2 KO E18.5 embryos and five control (four heterozygous and one WT) embryos. Electrical stimulation (1 or 20 Hz) of the phrenic nerve stump resulted in clearly visible contraction of the hemidiaphragm muscle of all embryos tested, demonstrating successful neurotransmission at their NMJs (for video recordings, see Additional file 1). We measured spontaneously occurring miniature endplate potentials (MEPPs) at TGF-β2 KO NMJs and found no changes in their amplitude (2.16 ± 0.16 mV), 0–100% rise time (5.25 ± 0.69 ms) or frequency (1.47 ± 0.19/minute) compared to the controls (Figure 3a–c and Figure 3i, left panel). Similarly, 0–100% rise-time (3.54 ± 0.20 ms) and amplitude (31.15 ± 4.73 mV) of nerve stimulation-evoked endplate potentials (EPPs) at TGF-β2 KO NMJs were not statistically significantly different from the controls (Figure 3f–g,k), as was the calculated quantal content (14.34 ± 1.63; Figure 3h), that is, the number of transmitter quanta released upon one nerve impulse. Muscle fiber action potentials following from successful neurotransmission showed similar characteristics in TGF-β2 KO and control NMJs (Figure 3j). The delay between stimulation of the nerve stump and the start of the postsynaptic response (either a muscle action potential or an EPP, depending on the actual resting membrane potential of the muscle fiber) was 6.04 ± 0.08 ms, which was not different from control (Figure 3e), indicating a normal action potential velocity at TGF-β2 KO intramuscular motor axons and a normal speed of synaptic transmission. We evoked acetylcholine release by the secretagogue α-latrotoxin. The MEPP frequency at both TGF-β2 KO and control NMJs rose to similarly high levels of around 1,500/minute (Figure 3d and Figure 3i, right panel), showing a normal capability for sustained transmitter release at TGF-β2 KO NMJs. Overall, these electrophysiological analyses demonstrate normal pre- and postsynaptic function at the TGF-β2 KO NMJ.

Altogether, after analyzing the morphology and function of the NMJ, we found no alteration in embryonic TGF-β2 KO animals that could account for their inability to breathe and, thus, turned to the central rhythm generating network in the brainstem, the preBötC.

### Severe impairment of network activity and synaptic transmission in preBötC neurons in TGF-β2 KO mice

**Figure 4:**
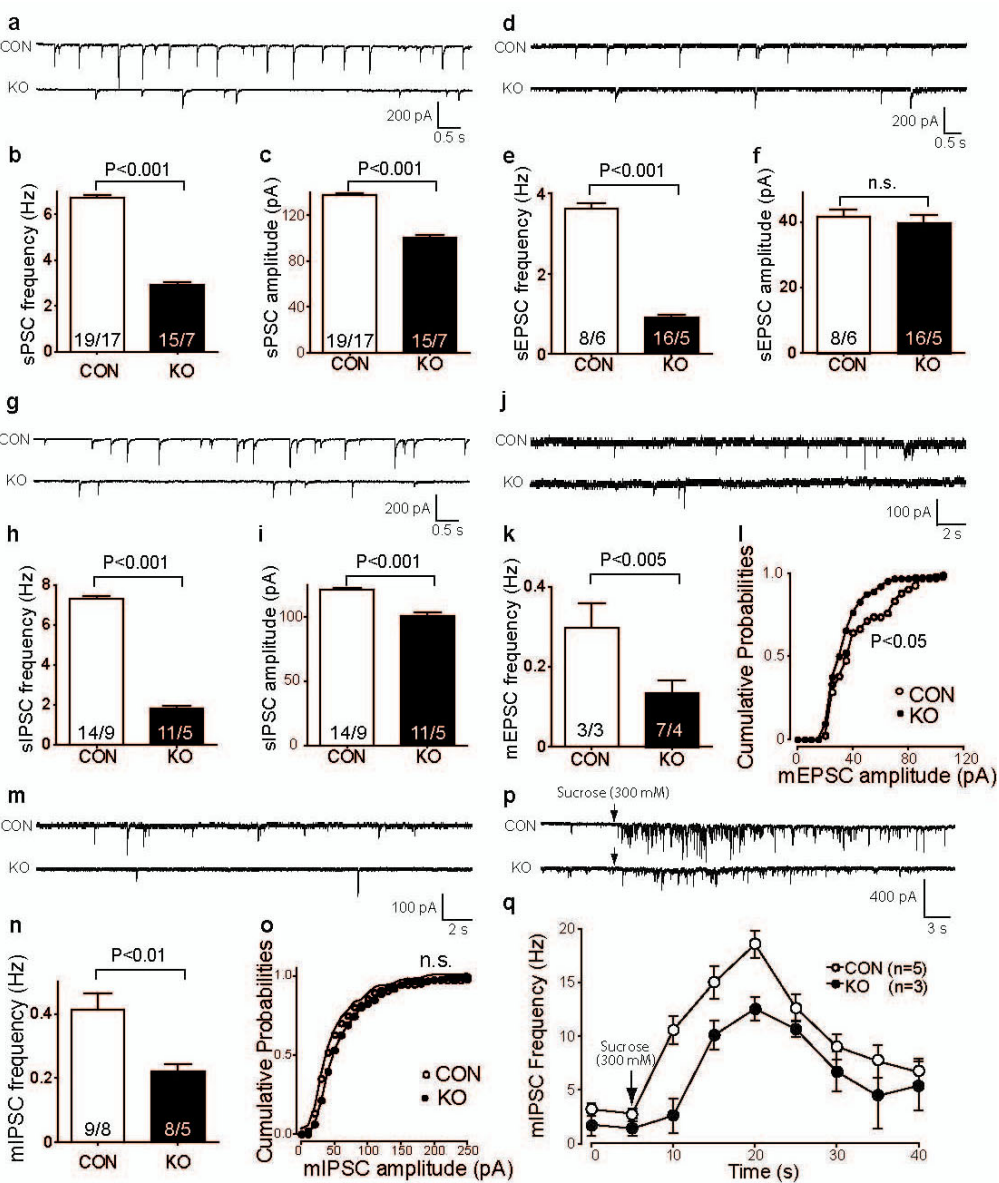
**Severe impairment of the overall network activity and of inhibitory and excitatory synaptic transmission in pre-Bötzinger-complex (preBötC) neurons of transforming growth factor (TGF)-β2 knock-out (KO) mice. **(a-c)Representative recordings (a), mean frequency (b) and amplitude (c) of spontaneous network activity (total spontaneous postsynaptic currents (sPSCs)) in brainstem preBötC neurons. (d-f) Representative recordings (d), mean frequency (e) and amplitude (f) of pharmacologically isolated spontaneous GABA/glycinergic postsynaptic currents (sIPSCs) in brainstem preBötC neurons. (g-i) Representative recordings (g), mean frequency (h) and amplitude (i) of spontaneous glutamatergic postsynaptic current (sEPSCs) in brainstem preBötC neurons. (j-l) Representative recordings (j), mean frequency (k) and amplitude (l) of pharmacologically isolated miniature GABA/glycinergic postsynaptic current (mIPSCs) in brainstem preBötC neurons. (m-o) Representative recordings (m), mean frequency (n) and amplitude (o) of pharmacologically isolated miniature glutamatergic postsynaptic currents (mEPSCs) in brainstem preBötC neurons. (p) Representative traces of mISPCs (p) in response to application of 300 mM sucrose (arrow) in wild-type (WT) and KO mice. (q) Time-frequency relationship of mIPSCs evoked by hyperosmotic sucrose stimulation. Results are given as mean ± standard error of the mean and numbers in bars indicate the number of tested neurons/mice for each genotype. CON, control.

The preBötC contains the kernel of the central respiratory rhythm generating network [30]. This network needs to be fully functional at the time of birth and, therefore, serves as an ideal model to study synaptogenesis and functional synaptic maturation in the brain at perinatal stages. To test whether functional failures in the brainstem respiratory network might cause the severe phenotype in TGF-β2 KO mice, we analyzed spontaneous excitatory and inhibitory synaptic transmissions of neurons in the preBötC area. We first monitored total spontaneous postsynaptic currents (sPSCs) using whole-cell recording in preBötC neurons. These currents represented the general activity level of the respiratory network in the preBötC. We found that both the frequency and amplitude of sPSCs in preBötC neurons were largely diminished in TGF-β2 KO mice (frequency: -56%; amplitude: -27%; Figure 4a–c). Thus, in TGF-β2 KO mice the general network activity was severely depressed compared to their littermates.

To determine whether the loss of TGF-β2 preferentially impaired inhibitory or excitatory synaptic transmission, we further analyzed pharmacologically isolated spontaneous GABA/glycinergic postsynaptic currents (sIPSC) and glutamatergic postsynaptic currents (sEPSC) in KO mice and their control littermates. The frequency of sEPSCs in preBötC neurons was strongly reduced (-75%), whereas the amplitude of sEPSCs was not significantly changed in KO mice (Figure 4d–f). In contrast, both the frequency and amplitude of sIPSCs in preBötC neurons were decreased in TGF-β2 KO mice (frequency: -75%; amplitude: -17%; Figure 4g–i). In summary, both inhibitory and excitatory contribution to the network activity within the preBötC was strongly affected after the loss of TGF-β2.

To determine the gene-deletion related pre- and postsynaptic changes, we further analyzed miniature GABA/glycinergic postsynaptic currents (mIPSC) and miniature glutamatergic postsynaptic currents (mEPSC) in preBötC neurons in the presence of tetrodotoxin (TTX; 0.5 μM). Here, the frequency of mEPSCs was severely depressed (-54%), whereas the amplitude of mEPSCs was only moderately decreased in KO mice (-19%; Figure 4j–l). The frequency of mIPSCs in preBötC neurons was significantly decreased in TGF-β2 KO embryos (-47%), while there was no significant difference in the amplitudes of mIPSCs between all littermates (Figure 4m–o). The reductions of the frequencies of mIPSCs could be caused by presynaptic defects. To further confirm this, we evoked mIPSCs by the application of hypertonic extracellular medium containing 300 mM sucrose. This treatment triggers the release of all fusion competent synaptic vesicles, the number of which is a key determinant of the rates of miniature events [31]. The frequency of mIPSCs and the total charge transfer induced by hypertonic stimulation were reduced in TGF-β2 KO embryos (Figure 4p–q). These data together suggest that the deletion of TGF-β2 mainly impaired the presynaptic component of both inhibitory and excitatory synaptic transmission.

### Synaptic protein expression in TGF-β2 KO mice

**Figure 5:**
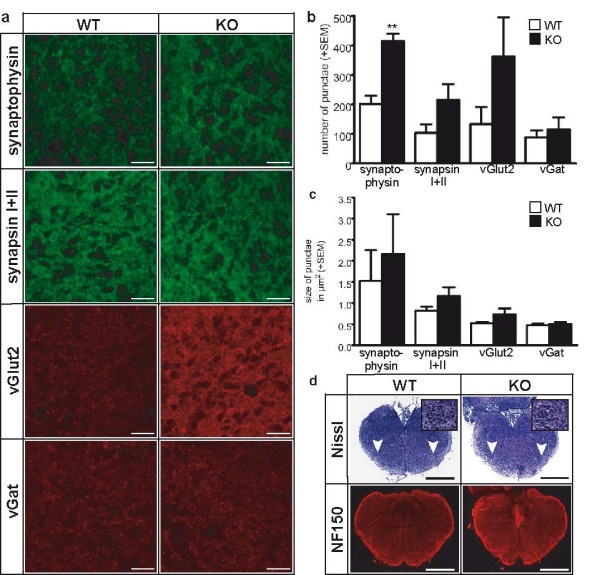
**Immunohistochemical staining, synaptic number and size of neurons in the pre-Bötzinger-complex (preBötC) area.** (a) Immunohistochemical staining of pre- and postsynaptic marker proteins of synapses in the preBötC area in wild-type (WT) and knock-out (KO) brain sections. Scale bar, 20 μm. (b) Quantification of positive punctae in WT and KO animals in the preBötC area. (c) Size of positive punctae in WT and KO animals in the preBötC area. (d) Nissl-stain to identify sections containing the nucleus ambiguus (arrowhead) and staining with anti-neurofilament (NF)150-antibody in WT and KO brain sections. Insets show higher magnifications of the nucleus ambiguus that were used to identify the region of the preBötC. Scale bar, 500 μm. SEM, standard error of the mean. **p < 0.01

One possible explanation for the extensive reduction of the frequencies of glutamatergic or GABA/glycinergic miniature events in TGF-β2 KO embryos may be a reduced number of the corresponding synapses. To test this possibility, we analyzed the number and size of synapses in the preBötC area in E18.5 animals. Synapses were labeled immunohistochemically with antibodies against general presynaptic proteins (synaptophysin and synapsin I+II) and markers for glutamatergic or GABAergic synapses (vGlut2 and vGat, respectively). Staining revealed a punctate staining for all markers used as shown in Figure 5a. Unexpectedly, quantitative determination showed that the number of synaptophysin-positive punctae was significantly increased and also synapsin I+II- and vGlut2-positive punctae tended to be increased in TGF-β2 KO mice compared to WT littermates (Figure 5b). The number of vGat-positive punctae remained unchanged. Also, the size of synaptophysin-, synapsin I+II-, and vGlut2-positive punctae tended to be increased in TGF-β2 KO brains compared to WT mice (Figure 5c).

We also analyzed protein samples from brainstems of embryonic (E18.5) WT and TGF-β2 KO animals on western blots to compare the amounts of several other synaptic proteins. The levels of the general, excitatory and inhibitory pre- and postsynaptic proteins from WT and KO brainstems tested are shown in Table 1. We observed changes in the amount of several synaptic proteins, although none of them were statistically significant as the variation within groups was rather high.

**Table 1:** Expression levels of presynaptic and postsynaptic proteins in transforming growth factor-β2 knock-out mice

	Expression level	*P*-value
Presynaptic proteins		
vGat	93 ± 2	NS
vGlut1	208 ± 101	NS
vGlut2	80 ± 11	NS
Synaptotagmin	79 ± 8	NS
Synaptobrevin	124 ± 16	NS
Munc13-1	184 ± 40	NS
		
Postsynaptic proteins		
Gephyrin	114 ± 59	NS
Neuroligin-1*	101 ± 9	NS
Neuroligin-2	84 ± 23	NS
Neuroligin-3	90 ± 11	NS
NMDAR*	152 ± 23	NS
GlyR*	107 ± 12	NS

Protein levels in postnuclear fraction of brainstem material of embryonic day 18.5 knock-out (KO) embryos, given as percentage of wild-type (WT) levels ± standard error of the mean. WT and KO, n = 4; *WT, n = 4; KO, n = 3. NS, not significant.

Histological analysis of the brainstem in WT and TGF-β2 KO embryos revealed no difference in its overall structure (as illustrated by Nissl-staining) and also the fiber connections, as illustrated with anti-neurofilament 150 kDa antibody, did not show alterations in the TGF-β2 KO animals (Figure 5d).

### Ultra-structural analysis of synapses of preBötC neurons reveals no changes in TGF-β2 KO mice

**Figure 6:**
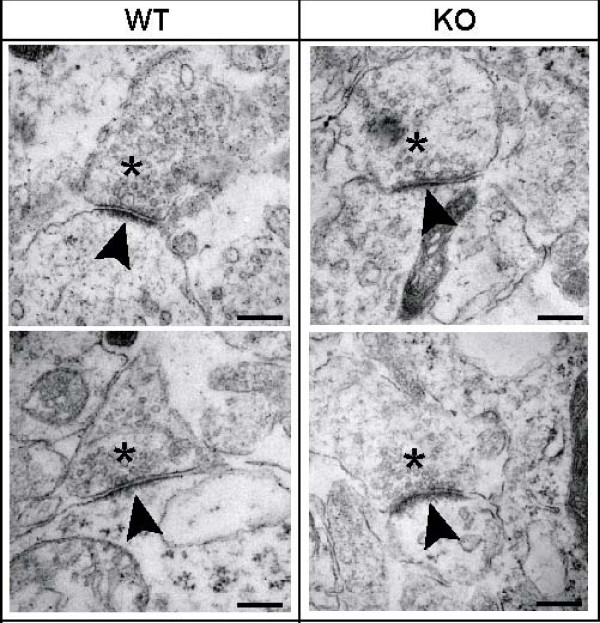
**Ultra-structural analysis of synapses in the brainstem of wild-type (WT) and transforming growth factor (TGF)-β2 knock-out (KO) mice at embryonic day 18.5.** Synapses of WT and TGF-β2 KO neurons in the pre-Bötzinger-complex area exhibit presynaptic vesicles (asterisks), a synaptic cleft and a distinct postsynaptic density (arrowheads). Scale bar, 250 nm.

We also analyzed the ultra-structure of synapses in the preBötC area and found that both WT and KO neurons formed synapses that contained presynaptic vesicles, large dense core vesicles and docked vesicles (Figure 6). Different stages of synaptic differentiation were present and difficult to compare. Therefore, we concentrated only on the structure of the most mature synapses. Both WT and KO synapses displayed comparable synaptic clefts, postsynaptic densities and similar densities of synaptic vesicles in the vicinity of the presynaptic membrane. Thus, these data indicate that the pronounced functional synaptic phenotype in TGF-β2 KO mice can neither be simply explained by reduced numbers of synapses nor by synaptic malformations.

## Discussion

We have examined the role of TGF-β2 in the development and function of peripheral and central synapses *in vivo *by carefully analyzing TGF-β2 KO mice. The present data show that TGF-β2 is an important regulator of proper synapse and neuronal network function within the central nervous system. We conclude this from the fact that its loss resulted in a dramatic decrease of both inhibitory and excitatory synaptic transmission in the respiratory neuronal network of the preBötC, one of the earliest neuronal networks established. In contrast to this important synaptic role of TGF-β2 in the central nervous system, we show that its loss alters neither synapse morphology nor function at NMJs in the periphery. Therefore, disturbances in the peripheral execution of breathing were excluded as the defect underlying the inability to breathe, and we rather suggest that the perinatal death of TGF-β2 KO mice is due to a malfunction in their central respiratory center.

At perinatal stages, the brainstem respiratory rhythm-generating network, of which the preBötC is part, is functionally more mature than other neuronal networks of the brain such as the hippocampus or the cortex. Our data clearly demonstrate that TGF-β2 plays an essential role in the functional maturation of both excitatory and inhibitory synapses within this network. Nevertheless, the widespread distribution of TGF-β2 in the developing and postnatal nervous system and the severity of the impaired synaptic transmission strongly argue for a general role of TGF-β2 in the regulation of synaptic function.

### Synaptogenesis and synaptic function

The rationale to study TGF-β2-dependent synaptogenesis came from the observation that TGF-β2 KO mice die from congenital cyanosis. As other plausible causes such as cardiovascular or pulmonary failure had already been excluded by others [12], we followed the hypothesis that a neuromuscular defect was the cause of death. In that context, two possible scenarios could be envisioned: impaired peripheral innervation due to aberrant morphology or dysfunction of the NMJ; or impaired central regulation of breathing.

Evidence arising from the analyses of the invertebrate NMJ revealed that the *Drosophila *BMP homologue gbb acts as a retrograde signal in synaptogenesis [20,21] and is thought to provide positional cues to guide synapse development. Mutations of the BMP type II receptor wit show inhibited target-dependent synapse formation in the larva, smaller bouton numbers and defects in neurotransmitter release at the *Drosophila *NMJ [22,23]. These results prompted us to analyze the NMJs of TGF-β2 KO embryos, although it should be kept in mind that despite their similar expression pattern at the NMJ, wit is a BMP type II receptor orthologue and gbb also represents a BMP orthologue rather than a TGF-β orthologue, making it a significant surprise if mammalian TGF-β carried invertebrate BMP functions.

The morphological analyses of NMJs of TGF-β2 KO diaphragms revealed a 25% reduction in the number of NMJs (Figure 2g) while the innervation pattern and NMJ shape remained unaltered (Figure 2a–f). The remaining NMJs, however, were not functionally impaired, which can be deduced from the observed muscle contraction after nerve stimulation and the observations that neither spontaneously occurring miniature endplate potentials, nerve stimulation-evoked endplate potentials nor muscle fiber action potentials showed differences when compared to the control group. The reduction of NMJs could be related to a synapse-independent malformation of the diaphragm muscle, which can be observed in TGF-β2 KO diaphragms (see disorganized muscle fibers in TGF-β2 KO diaphragms in the video, which is presented as Additional file 1). The reduction of AChR clusters could therefore be a muscle-intrinsic problem, as it is known that TGF-β does affect the differentiation of muscle tissue [32-34].

Analyses of the central regulation of breathing were performed at the level of the respiratory rhythm generating brainstem network. To ensure the most vital function in life, the respiratory network is one of the most robust neuronal networks in the brain, which is able to adapt to most disturbances during life. We show for the first time that both spontaneous and miniature IPSCs and EPSCs were significantly depressed in TGF-β2 KO embryos (Figures 4d–i). This reflects reduced overall network activity (Figures 4a–c). It is also quite striking that although the frequencies of both mEPSCs and mIPSCs were severely diminished, the amplitudes of the miniature events were not or only moderately decreased (Figures 4j–l).

As the overall structure within the brainstem network was not altered (Figure 5d), the loss in the frequency of sPSCs could be interpreted either as a reduced number of excitatory or inhibitory synapses in the brainstem preBötC region or as an impairment of the transmitter release machinery in all synapses. When we tested the number of synapses by counting punctae that were positively stained for presynaptic markers, we did not observe a reduction, but rather a tendency towards an increase (Figure 5b). Also, the western blot analysis of different synaptic proteins yielded no evidence of a lack of synaptic equipment, as almost all proteins tested were again rather increased in their expression levels (Table 1). The amount of postsynaptic neurotransmitter receptor protein, such as NMDAR or GlyR, was not reduced (Table 1), which argues against a possible lack of postsynaptic sensitivity. Previously, neuroligins have been shown to be required for proper synapse maturation and brain function [35]. A phenotype closely resembling that of TGF-β2 KO mice was described for the neuroligin 1–3 triple KO. Neuroligin 1 is specifically localized to glutamatergic postsynaptic specializations, whereas neuroligin 2 is localized to inhibitory synapses [36]. However, when analyzing protein levels of synaptic components, neuroligin expression was not reduced in TGF-β2 KO mice, suggesting that TGF-β2 does not determine synapse function via regulation of neuroligins.

It is possible that the system either tries to compensate for non-functional synaptic sites by generating more contacts or that it fails to eliminate redundant or non-functional synapses. In fact, normal synaptic transmission is essential for the elimination of synapses [37]. Thus, our data argue for a defective rather than reduced central synaptogenesis in mutant mice.

Our data demonstrate that the loss of TGF-β2 diminished both synaptic excitation and inhibition. As both GABA/glycinergic inhibition and glutamatergic excitation are essential for respiratory rhythm generation, the severe changes in synaptic inhibition and excitation most probably explain the lethal phenotype of TGF-β2 KO mice. Interestingly, the lack of TGF-β2 cannot be compensated for by other TGF-β isoforms during the embryonic phase either in excitatory or in inhibitory central synapses. This is highly intriguing as TGF-β2 and TGF-β3 have overlapping expression patterns in the brain as they are both expressed by glial cells and pyramidal neurons of the cortex (layers 2, 3, and 5) and the hippocampus, by magnocellular neurons of septal nuclei and the hypothalamus and all somato- and visceromotor neurons of the brainstem and the spinal cord [38]. Moreover, the isoforms share >97% homology [4] but seemingly cannot fulfill the same function in this context.

### TGF-β isoform specificity

As mentioned before, our results demonstrating the requirement for TGF-β2 for central neuronal network function are not only highly surprising with regard to the regulation of synapse function but also from a growth factor point of view. TGF-βs show a widespread distribution and overlapping expression. At least in most *in vitro *experiments TGF-β isoforms are indistinguishable from each other in their biological response. It is therefore highly surprising to see such a unique and dramatic phenotype for TGF-β2 KO mice *in vivo*. Comparison of our findings with the work on BMPs in *Drosophila *shows that apparently TGF-β2 does not have the same function as the BMP-7 homologue gbb, which we first expected from their comparable expression patterns at the NMJ. Another example of distinct functions for BMP-7 and TGF-β2 has been described for their role in the regulation of AMPA and kainate receptors in the human retina [39]. One should also take into account that the invertebrate NMJ is glutamatergic and does not use acetylcholine as mammalian NMJs do, which might explain why we found alterations in central but not in the peripheral synapses. The obvious difference in the roles of TGF-β2 at central and peripheral synapses might also be explained by a different capacity of the two systems to compensate for the loss of TGF-β2. Future experiments will dissect the evolution of synaptogenesis and regulation of synapse function within the diverging TGF-β superfamily.

### Mechanism of TGF-β2 mediated dysfunction

Having shown that lack of TGF-β2 led to impaired synaptic transmission, the question arises as to the mechanism by which an extracellular signaling molecule may regulate synaptic function. As both inhibitory and excitatory central synaptic transmission was depressed, TGF-β2 must affect general synaptic functions rather than neurotransmitter-specific aspects. Moreover, the analysis of miniature events and hypertonic sucrose stimulation pointed to a presynaptically localized defect in TGF-β2 KO embryos.

Evidence from the analysis of isolated *Aplysia *ganglia [9] suggested that TGF-β may act via phosphorylation of synapsin in mice and we indeed observed an influence of TGF-β on the phosphorylation of synapsin in hippocampal neurons (unpublished observation). Synapsin phosphorylation is a crucial event for the dissociation of synapsin from synaptic vesicles during normal vesicle cycling [40] and the subsequent release of synaptic vesicles from the actin-bound reserve pool into the readily releasable pool of vesicles. A mechanism involving the phosphorylation of synapsin has been shown for brain-derived neurotrophic factor [41] and was also discussed for the *chordin*^-/- ^mouse mutant, which has increased BMP signaling [42]. Nevertheless, synapsin is far from the only protein shared by the release machinery of excitatory and inhibitory terminals and further experiments will have to unravel the molecular targets of TGF-β2 at the synapse.

## Conclusion

Our results demonstrate for the first time that TGF-β2 influences the function of central synapses, rather than initial synapse formation and synaptogenesis in mice. We suggest that the functional alterations in the respiratory center of the brain are probably the underlying cause of the perinatal death of the TGF-β2 KO mice.

## Materials and methods

### Animals

*TGF-β2*^+/- ^mice were offspring of breeding pairs kindly provided by Tom Doetschman, University of Cincinnati (Cincinnati, Ohio, USA), and were kept in the mouse facility of the department. To obtain TGF-β2 KO animals, *TGF-β2*^+/- ^mice were mated overnight and the day on which females had a vaginal plug was defined as E0.5. Genotyping was performed from tail biopsies as described elsewhere [12]. All electrophysiological analyses were performed on diaphragms or brainstem neurons of mice whose genotype was unknown to the experimenter. As no significant difference between WT and heterozygous animals has been described, *TGF-β2*^+/- ^mice and *TGF-β2*^+/+ ^mice were grouped into a control group for all electrophysiological experiments.

### Whole-body plethysmography

Breathing behavior of embryonic (E18.5) TGF-β2 KO mice and WT littermates was recorded by whole-body plethysmography. After caesarean section, unanesthetized pups were directly transferred into a closed 15 ml chamber, which was connected to a differential pressure transducer (CD15 Carrier Demodulator, ValiDyne, Northridge, CA, USA). The analogue signal of ventilation-related changes in air pressure was amplified and digitized using an A/D-converter (DigiData 3200, Axon Instruments, Union City, CA, USA) and analyzed using commercially available AxoTape and AxoGraph software (Axon Instruments).

### Diaphragm whole-mount preparation, immunohistochemistry and confocal microscopy

Diaphragms were dissected from embryonic mice and fixed in 4% paraformaldehyde (PFA) for 2 h at 4°C between two glass slides. After three washes in phosphate-buffered saline (PBS), the tissue was permeabilized in 0.5% Triton-X/PBS for 30 minutes, followed by the blocking of unspecific binding sites in 10% normal goat serum/PBS. Incubation with the primary antibody (1:500 rabbit anti-neurofilament 150 kDa, Chemicon/Millipore, Livingston, UK; or 1:500 rabbit anti-S100, Dako, Hamburg, Germany; both diluted in blocking solution) was carried out overnight at room temperature. Diaphragms were then washed three times in PBS and incubated with the secondary antibody (1:500 goat-anti-rabbit-IgG-Cy3, Jackson Immuno Research/Dianova, Hamburg, Germany) plus FITC-labeled α-bungarotoxin (1:500; Molecular Probes, Leiden, The Netherlands), both diluted in PBS. After a final washing, diaphragms were flat-mounted in mounting medium (Dako).

Images were acquired with a confocal laser-scanning microscope (LSM SP2, DM IRE2, Leica) in a blinded manner. Pictures were taken at different magnifications (200×, 400×, or 630×) with oil-immersion lenses as 2 μm stacks and gain and offset were kept constant for all specimens of one experiment. The pinhole was set to 80 μm. For comparison between WT and KO diaphragms, stacks were merged in a maximum projection. To determine the branching pattern of the phrenic nerve, processes were analyzed as described by others [28]. Briefly, equidistant lines (20 μm) were drawn parallel to the main trunk and the number of crossing branches was determined. The overall number of branches and bifurcations on either side of the main trunk was determined for statistical comparison.

The number of AChR clusters was determined by touch counting and is given as the mean of five fields on each semi-diaphragm.

### Electrophysiological recordings at the neuromuscular junction

Synaptic transmission at NMJs of three TGF-β2-KO and five control (one WT and four heterozygous) E18.5 embryos was assessed electrophysiologically with *ex vivo *intracellular microelectrode measurements. The values of all studied synaptic parameters of the WT control fell within the range of values obtained at the heterozygous controls. Therefore, all values were pooled into one control dataset.

Diaphragm muscle was dissected and mounted in Ringer's medium (116 mM NaCl, 4.5 mM KCl, 2 mM CaCl_2_, 1 mM MgSO_4_, 1 mM NaH_2_PO_4_, 23 mM NaHCO_3_, 11 mM glucose, pH 7.4, pre-bubbled with 95% O_2_/5% CO_2_) at 26–28°C. Muscle fibers were impaled at the NMJ region with an approximately 20 MΩ glass capillary micro-electrode, connected to a Geneclamp 500B amplifier (Axon Instruments/Molecular Devices). Signals were digitized, stored and analyzed (off-line) on a PC using a Digidata 1322A interface, Clampex 9 and Clampfit 9 programs (Axon Instruments/Molecular Devices) and MiniAnalysis 6 (Synaptosoft, Decatur, GA, USA).

Intracellular recordings of MEPPs, the spontaneous depolarizing events due to uniquantal acetylcholine release, were made at several different NMJs within the muscle. The phrenic nerve stump was stimulated supramaximally via a suction electrode at 1 and 20 Hz. The resulting muscle contraction of the hemidiaphragm was visually monitored and example contractions were recorded on videotape. Examples of a muscle fiber action potential resulting from a single nerve stimulus were recorded. To be able to record evoked synaptic responses (EPPs), muscle fibers were allowed to depolarize to -20 to -30 mV (which normally occurs quickly after impalement, presumably due to a damaging effect of microelectrode impalement to the relatively thin embryonic muscle fibers). This leads to inactivation of Na^+ ^channels, so that a muscle action potential no longer occurs and the underlying EPP could be recorded.

The amplitudes of EPPs and MEPPs recorded at each NMJ were linearly normalized to -75 mV resting membrane potential. From the grand-mean values of each muscle, the number of acetylcholine quanta released per nerve impulse, that is, the quantal content, was calculated by dividing the mean EPP amplitude by the mean MEPP amplitude.

MEPPs were also recorded after application of 2.5 nM α-latrotoxin (Alomone Laboratories, Jerusalem, Israel). In these experiments, TTX (1 μM, Sigma-Aldrich, Zwijndrecht, The Netherlands) was added to block muscle action potentials, either occurring spontaneously or triggered by superimposed high frequency MEPPs. All electrophysiological NMJ data are given as group mean values ± standard error of the mean with n as number of muscles per group and 3–20 NMJs sampled per muscle. Statistical significance was tested with Student's *t*-test.

### Electrophysiological recordings in brainstem slices

Acute slices containing the preBötC from embryonic littermate mice (E18.5) were used for whole-cell recordings as described earlier [43]. Briefly, the bath solution in all experiments consisted of 118 mM NaCl, 3 mM KCl, 1.5 mM CaCl_2_, 1 mM MgCl_2_, 25 mM NaHCO_3_, 1 mM NaH_2_PO_4_, 5 mM glucose, pH 7.4, aerated with 95% O_2 _and 5% CO_2 _and kept at 28°C. The pipette solution for patch-clamp-recordings contained 140 mM Kgluconate (glutamatergic PSCs) or 140 mM KCl (GABA/glycinergic PSCs), 1 mM CaCl_2_, 10 mM EGTA, 2 mM MgCl_2_, 4 mM Na_3_ATP, 0.5 mM Na_3_GTP, 10 mM HEPES pH 7.3. Spontaneous GABA/glycinergic and glutamatergic PSCs (sIPSCs and sEPSCs) were recorded from neurons of the preBötC in the presence of 10 μM CNQX ((6-cyano-7-nitroquinoxaline-2,3-dione) or 1 μM strychnine and 1 μM bicuculline, respectively. Spontaneous mIPSCs and mEPSCs were recorded as described above, but in the presence of 0.5 μM TTX. In order to elicit a hypertonic response (Figure 4p–q), sucrose (300 mM) was directly applied in close proximity to neurons by glass pipettes. To minimize the variation between experiments, tip size of the pipette, pressure (0.5 mbar) and time (500 ms) were kept constant for all experiments. In addition, the distance between pipette tips and the cell were monitored using a LCD camera, and was also kept constant between different experiments. Generally, signals with amplitudes at least two times above the background noise were selected, and the statistical significance was tested in each experiment. In all animals tested, there were no significant differences between the noise levels between different genotypes. All postsynaptic currents recorded were amplified and filtered by a four-pole Bessel filter at a corner frequency of 2 kHz, and digitized at a sampling rate of 5 kHz using the DigiData 1200B interface (Axon Instruments). Data acquisition and analysis were carried out using commercially available software (pClamp 9 and AxoGraph 4.6, Axon Instruments, and Prism 4 Software, GraphPad, La Jolla, CA, USA).

### Immunohistochemistry and light microscopic quantification of synapses of preBötC neurons

Embryos (E18.5) were perfused transcardially with 0.5% or 4% PFA. Brains were dissected and post-fixed in the respective fixative for 48 h (0.5% PFA) or 1 h (4% PFA). Following cryoprotection with sucrose, brains were embedded in cryo-medium and frozen on dry ice. Serial frontal sections (14 μm) were collected and Nissl-stained to identify the nucleus ambiguus and subsequent sections were used for immunohistochemistry. After having been blocked with 10% goat serum/0.1% Triton-X, sections were stained for synaptophysin (1:80; Dako), synapsin I+II (1:100; Synaptic Systems, Goettingen, Germany), vGlut2 (1:8,000; Chemicon), and vGat (1:4,000; Chemicon), using the respective secondary antibodies (1:100 goat-anti-mouse-IgG-FITC, 1:100 goat-anti-rabbit-IgG-FITC, both Jackson Immuno Research/Dianova; 1:200 goat-anti-guinea pig-IgG-rhodamin, Chemicon).

Images were acquired with a confocal laser-scanning microscope (LSM SP2, DM IRE2, Leica) in a blinded manner. Single layer pictures were taken at a magnification of 630× (oil-immersion lens) and 2× digital zoom. The pinhole was set to 120 μm. Gain and offset were kept constant for all specimens of one experiment. For the analysis, images were imported into ImageJ software (NIH, US). To quantify positive punctae, a threshold was manually set for each image prior to binarization and a particle analysis to determine particle number and area.

### Ultra-structural analyses

For ultra-structural analysis, brainstem sections (200 μm) were prepared from embryonic (E18.5) WT and TGF-β2 KO animals and immersion-fixed with 3.75% acrolein, 2% PFA, 0.1 M MOPS, pH 7.0 for approximately 5 minutes and in 4% PFA, 0.1 M MOPS, pH 7.0 overnight. Subsequently, the tissue was processed according to standard procedures. Briefly, the tissue was osmicated in 1% OsO_4 _and flat-embedded in Spurr's epoxy medium. Ultra-thin sections were cut and stained with uranyl acetate and lead citrate before electron microscopic observation with a Zeiss Leo 906E.

### Protein analysis

Material for western blot analysis was obtained from brainstem tissue of embryonic (E18.5) mice. Brainstems were mechanically homogenized in homogenization buffer (320 mM sucrose, 5 mM HEPES, pH 7.4, 0.1 mM EDTA). The crude lysate was centrifuged (1,000 × g for 10 minutes) to obtain the post-nuclear fraction. Protein concentrations were determined using Biorad's Bradford reagent and equal amounts of protein (20 μg) were dissolved on 10% or 15% acrylamide gels. Proteins were blotted onto nitrocellulose-membranes (Biorad, Munich, Germany). Antibodies against pre- and postsynaptic proteins were used in the following concentrations: 1:10,000 vGlut1 (Chemicon); 1:7,500 vGlut2 (Chemicon); 1:5,000 vGat (Synaptic Systems); 1:500 synaptotagmin (Synaptic Systems); 1:7,500 synaptobrevin (Synaptic Systems); 1:5,000 neuroligin 1, 1:250 neuroligin 2, 1:250 neuroligin 3, 1:5,000 NMDAR (all neuroligin-antibodies and NMDAR-antibody from N. Brose, Goettingen, Germany); 1:500 GlyR (Synaptic Systems). Primary antibodies were detected by the respective horse-radish peroxidase (HRP)-conjugated secondary antibodies (Jackson Immuno Research/Dianova) and visualized using the enhanced chemiluminescence (ECL) method. Exposed X-ray films were scanned (Epson Twain software with Epson Perfection 1240U-scanner) and the intensity of bands was densitometrically measured using AlphaEaseFC-software (Alpha Innotech Corporation, Kasendorf, Germany). Blots were reprobed for GAPDH and signals of the synaptic proteins were normalized to the GAPDH signal.

### Statistical analysis and data illustration

All experiments were carried out with material from at least three animals per group or from three independent experiments. Values from the immunohistochemical analyses were imported into GraphPad Prism 4.0 software and tested for statistical significance using the Student's *t*-test. Differences were considered significantly different when *P *< 0.05.

All electrophysiological data are expressed as mean ± standard error of the mean. *P*-values represent the results of two-tailed unpaired Student's *t*-test with Welch's correction. The data of mIPSCs and mEPSCs were tested with a Kolmogorov-Smirnov test (MiniAnalysis, Synaptosoft, Inc., Decatur, GA, USA).

Graphs were designed with GraphPad Prism 4.0 software and figures were prepared with Adobe Photoshop 6.0 software.

## Abbreviations

AChR: acetylcholine receptor; BMP: bone morphogenetic protein; E: embryonic day; EPP: endplate potential; gbb: glass-bottom-boat; KO: knock-out; mad: mothers against decapentaplegic; med: medea; MAPK: mitogen-activated protein kinase; MEPP: miniature endplate potential; mEPSC: miniature glutamatergic postsynaptic current; mIPSC: miniature GABA/glycinergic postsynaptic current; NMJ: neuromuscular junction; PBS: phosphate-buffered saline; PFA: paraformaldehyde; preBötC: pre-Bötzinger-complex; sax: saxophone; sEPSC: spontaneous glutamatergic postsynaptic current; sIPSC: spontaneous GABA/glycinergic postsynaptic current; sPSC: spontaneous postsynaptic current; TGF: transforming growth factor; tkv: thickveins; TTX: tetrodotoxin; wit: wishful thinking; WT: wild type.

## Competing interests

The authors declare that they have no competing interests.

## Authors' contributions

KH carried out the plethysmography measurements, all immunohistochemical and immunoblot experiments, and participated in drafting the manuscript. VS carried out plethysmography measurements and performed and analyzed the electrophysiological experiments of the brainstem. JP performed the functional analyses of the NMJ of the diaphragm. MR carried out the electron microscopy analysis. FV participated in the immunoblot analyses. WZ participated in the electrophysiological recordings and in the analysis and interpretation of the data. KK conceived the study, designed and coordinated it and drafted the manuscript. All authors read and approved the final manuscript.

## Supplementary Material

Additional file 1 Phrenic nerve stimulation in transforming growth factor-β2 knock-out and control diaphragms. Video recordings of nerve stimulation-evoked muscle contraction in transforming growth factor-β2 knock-out and control embryonic day 18.5 hemidiaphragms at stimulation frequencies of 1 and 20 Hz. Click here for file
